# Digital Tools to Support Postpartum Recovery: A Systematic Review

**DOI:** 10.1177/26884844251380146

**Published:** 2025-09-17

**Authors:** Adam J.E. Kwasnicki, Chiara Rizk, Max J. Western, Hasan Zaidi, Richard M. Kwasnicki

**Affiliations:** ^1^ University of Bath, Bath, UK.; ^2^ Imperial College London, London, London, UK.

**Keywords:** digital technology, exercise, physical activity, postpartum

## Abstract

**Introduction:**

Returning to physical activity (PA) postpartum is challenging due to physical, psychological, and socio-cultural barriers. Successful return is associated with physical and mental health benefits. Advancements in digital technology access and a digital focus for providers offer potential areas to improve PA; however, current strategies and their efficacy have not been described in the literature.

**Methods and Analysis:**

A systematic review of studies evaluating digital technologies in returning postpartum women to PA was completed according to Preferred Reporting Items for Systematic Reviews and Meta-Analyses guidelines. Electronic databases: Web of Science, SCOPUS, Embase, APA Psycnet, and PubMed were searched from inception until 24 July 2022. Primary objectives were to return to PA postpartum when utilizing digital technologies, and secondary objectives included patient satisfaction and confidence towards returning to PA.

**Results:**

The review returned 14 eligible studies (*n* = 2714), using digital technologies such as pedometers, text messaging, and smartphone applications. Outcome measures were patient questionnaires, although some used activity trackers. Statistically significant differences in PA were seen in 7 studies with an average intervention increase of approximately 108%. Secondary outcomes of perceived reduction in barriers, increased satisfaction, and self-reported confidence towards engaging in PA were investigated in 6 of the studies, with 4 of the studies reporting an increase in these outcomes.

**Conclusion:**

Digital interventions may play a role in supporting return to PA after childbirth, particularly as part of a multi-modal strategy. However, further research randomizing participants into digital and standard arms is needed to quantify the specific contribution of digital tools.

## Introduction

Returning to physical activity (PA) postpartum can be a significant challenge. Physical challenges can be related to pain, such as pregnancy-related pelvic girdle pain,^1^ or pelvic floor pathology, such as stress incontinence.^2^ Factors can also be related to socio-cultural beliefs^3^ and scheduling challenges such as access to childcare and combining responsibilities with returning to work. A lack of knowledge and access to healthcare practitioners has also been identified as a barrier.^4^ Digital interventions alongside education and simple behavioral interventions could offer value in this area.

Improved levels of PA have positive associations with returning to a healthy weight,^5^ improved psychological wellbeing,^6^ and reducing risks of already high burden and prevalent diseases in the population, such as cardiovascular illness and diabetes.^7^ This can have positive consequences for both the individual and for the wider society.

As access to affordable digital technology improves and health systems prioritize digital health strategies,^8^ these tools may help address waiting list challenges by providing greater support strategies to enable an earlier return to activity. This is achieved through increasing patient confidence, and by reducing injuries requiring medical intervention. Various digital health applications are being used for musculoskeletal conditions and mental health support, among others,^9^ with opportunities for translation into the postpartum cohort.

This systematic review investigates the use of digital health technologies to assess their value in helping postpartum women return to PA. Secondary outcomes investigate the effect on patient quality of life and self-perceived ability, or confidence, in their ability to engage in PA during the postpartum period. Knowledge of this may help direct further research and resources into developing sustained and scalable strategies utilizing digital tools in the post-partum setting, along with all the personal and societal benefits that could be achieved.

## Methods

This systematic review has been registered with the International Prospective Register for systematic reviews (PROSPERO),^10^ adhering to a prespecified protocol and the Preferred Reporting Items for Systematic Reviews and Meta-analyses statement.^11^

### Identification of studies

This systematic review included studies on digital technologies currently used to assist postpartum adult women in returning to PA. For this study, all digital strategies (not conventional face-to-face interaction or paper handouts) were considered. Returning to PA in any form was also considered to broaden the scope of the review. The search strategy was developed with a librarian, where appropriate and sufficient databases for the topic and key search terms (digital technology, postpartum, and return to PA) were derived and can be seen in the full search strategy. The following electronic databases were searched from inception until 24 July 2022: Web of Science, SCOPUS, Embase, APA Psycnet, and PubMed (inclusive of MESH search). Furthermore, the reference list of the selected studies was also hand-searched for additional relevant articles.

Full search strategy was as follows: “digital technolog*” OR “apps” OR “self-management” OR “wearable sensors” OR “support groups” OR “wearable tech*” OR “digital education*” OR “online health” OR “pelvic health gadgets” AND “postpartum” OR “postnatal” OR “childbirth” OR “post pregnancy” OR “new mother” OR “pelvic floor” OR “post caesarean” OR “kegel” AND “physical activ*” OR “exercis*” OR “sport*” OR “training” OR “running” OR “returning to hobb*” OR “physical movement” OR “physical performance” OR “athletic*”.

### Study selection process

**FIG. 1.**
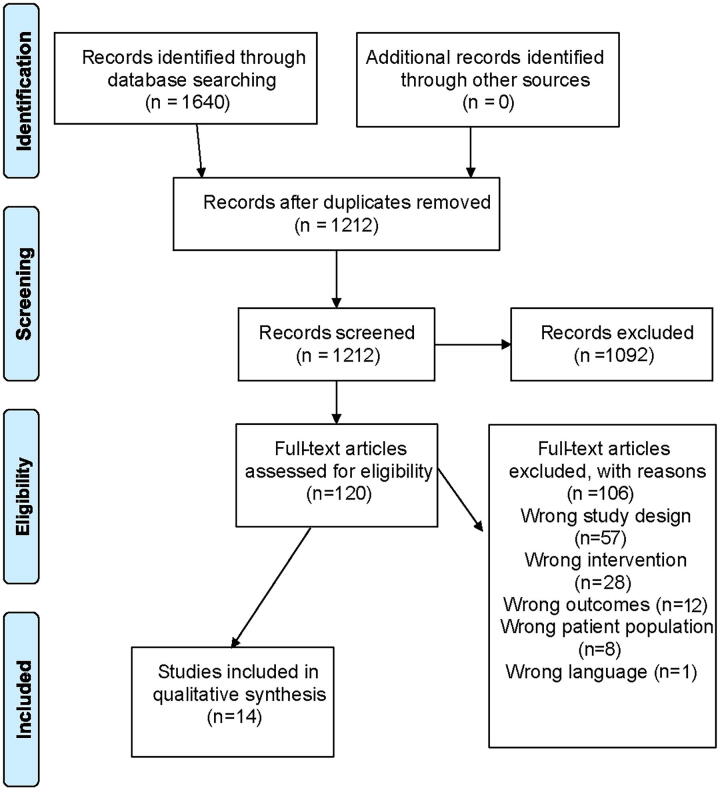
PRISMA flow chart with study selection process. PRISMA, Preferred Reporting Items for Systematic Reviews and Meta-analyses.

Search results were managed using Covidence software,^12^ which de-duplicated and allowed screening. Two independent reviewers (A.J.E.K. and C.R.) initially screened titles and abstracts against prespecified inclusion and exclusion criteria (Table 1). Full text screening was then completed by A.J.E.K. and C.R., to leave the remaining studies for data extraction. Any discrepancies were discussed with the senior author (R.M.K.), who had the final say (Fig. 1).

**Table 1.** Inclusion and Exclusion Criteria

Inclusion criteria	Exclusion criteria
English language studies	Studies on children (under 18)
Well described digital technologies	If there are significant co-morbidities or complications following birth
Return to physical activity	Not postpartum
Postpartum period of up to 12 months	No return to physical activity

### Quality assessment

The aim of the review was to investigate the evidence of how digital technologies can be used to support postpartum women back to PA. The Cochrane Risk of Bias 2 tool^13^ was used *via* Covidence software^12^ to determine the methodological quality of the included studies and their data (Table 2).

**Table 2.** Quality Assessment Data on Selected Studies

Study ID	Sequence generation	Allocation concealment	Blinding of participants and personnel	Incomplete outcome data	Selective reporting	Other sources of bias
Albright et al.^14^	N/A	N/A	N/A	High	Unsure	N/A
Albright et al.^15^	High	High	High	Low	Unsure	N/A
Bijlholt et al.^16^	High	Low	Low	High	Unsure	N/A
Burkart et al.^17^	Unsure	Unsure	Low	High	Unsure	N/A
Fjeldsoe et al.^18^	High	Low	Low	High	High	N/A
Kernot et al.^19^	High	High	Low	High	High	N/A
Khodabandeh et al.^20^	High	High	Low	High	High	Low
Kim et al.^21^	N/A	N/A	Low	High	Unsure	N/A
Lee et al.^22^	High	High	Unsure	Low	High	Unsure
Lewey et al.^23^	High	Unsure	Unsure	High	High	N/A
Lim et al.^24^	High	High	Low	High	Unsure	N/A
Maturi et al.^25^	High	Unsure	High	High	High	Unsure
Napolitano et al.^26^	High	Unsure	Unsure	Low	Unsure	Unsure
van der Pligt et al.,^27^	High	Unsure	Unsure	High	Unsure	Unsure

### Data extraction

Data were extracted using a prepared Excel spreadsheet^28^ developed specifically for the systematic review alongside the Covidence software data extraction tool (available on request). For each eligible study, the following data were extracted: digital technology used, protocol, type of study (including sample size), population, time postpartum when intervention utilized, length of intervention, primary PA outcome measures and effect, and any patient reported outcomes such as confidence and satisfaction, as well as study limitations (Table 3, more detail on interventions in Table 4).

**Table 3.** Data Extraction on Eligible Studies

Study ID	Sample size and population	Time after postpartum intervention started	Intervention length	Control	Primary PA outcome	Results	Secondary PA outcome	Limitations of study
Kernot et al., 2019^19^	Normal postpartum population App and pedometer- 4,1 Pedometer only- 39, Control- 40, Mean age- 31.8, > 1 child- 33%, Working %- 38	Mean 31.2 week	7.12 weeks	Written information only	Accelerometer derived MVPA Self-reported PA *via* Questionnaire	No significant difference between groups Accelerometer derived MVPA versus control 6 weeks: F = 0.21, *p* = .81; 6 months: F = 0.10, *p* = .91 Time spent walking compared to control 6 weeks: F = 0.61, *p* = 0.55; 6 months: F = 0.15, *p* = .90	QOL questionnaire No significant difference 62% felt the app was of interest 65% felt the app had assisted their increase in PA	Small sample size/effect size, homogenous group, lack of blinding
Kim et al., 2021^21^	With Gestational Diabetes Intervention- 57, Control- 62, Mean age- not presented- 47.9%, <35 > 1child-32.77% No data on work status	1 week	12 weeks	Standard care with written materials	Self-reported PA *via* questionnaire	significantly increased in intervention PA inc 0.89 ± 0.91, cont 0.23 ± 0.91 *p* < 0.001	N/A	Main focus of study on diet versus PA. Began 1 week after birth so PA challenges Only available on android.
Lewey et al., 2022^23^	Hypertensive disorders of pregnancy Intervention- 63 Control- 64 Mean age- 32.3 Working %- 25 Mean children- not reported	7.9 weeks	12 weeks	Wearable device, step count targets, SMS	Daily step count *via* accelerometer/pedometer	Difference in daily step count versus control, mean (SD), 647 (169–1124), *p* = 0.009	Difference in days achieving step goal 0.11 (0.04–0.19) *p* = 0.03	Both intervention and control arms had significant digital components
Lim et al., 2021^24^	With gestational diabetes Intervention- 101 Control- 99 Mean age- 32.5 Working %- 81.5 1 child- 46% 2 children- 37.6% 3 ± 16.4%	0.14 weeks	17.33 weeks	Standard care	Mean step count *via* accelerometer	Not compared versus control but: 4065 (95% CI 3483.6 to 4645.7) at 6 weeks. The mean step count increased to 4880 (95% CI 4195.4 to 5565.5) at 4 months (*p* = .04)	Health directed behavior scores: 0.16, 95% CI, *p* = 0.004 to 0.32	Limited data on PA. Not appeared to be compared to control. Primary focus of study on weight and diet. Intervention starts immediately postpartum which might not fit with PA
Maturi et al., 2011^25^	Physically inactive or low physical activity mothers Intervention- 32 Control- 34 Intervention mean age- 25.7 Control mean age- 24.8 Intervention working %- 21.9 Control working %- 23.5 Intervention no. of children- 1–87.5% Control no. of children- 1–91.2%	Mean 12.7–12.84 wee	12 weeks	Routine care	IPAQ Questionnaire for PA and step count	iPAQ Intervention Mod activity pre 5 (15.6), post 11 (33.4), Cont pre 7 (20.6), post 8 (23.5), Vig activity pre 4 (12.5), post 21 (65.6), Cont pre 4 (11.8), post 11 (32.5) difference between groups *p* = 0.001 Step count/day-only intervention group 9960 (2564)	N/A	Self-reported recordings
Napolitano et al., 2021^29^	Postpartum African American/Black women Intervention- 65 Control- 71 Mean age = 27.8 No. of children- >1 76.5% No data on working %	0.43 weeks	12 weeks	Usual care including written material	MVPA *via* questionnaire	No statistically significant change versus control Weekly MVPA Int- Pre 279.9 Post 618.4 *p* = 0.004 Change- Int 338.5 versus 81.1 control *p* = 0.10	Exercise self-efficacy- 13.7 to 14.2, *p* = 0.73	Started very early postpartum for PA Multiple outcomes measure with small sample size. Significant drop outs.
Albright et al., 2014^15^	Normal postpartum population Intervention- 154 Control- 157 InterventionMeanage-31.6 ControlMeanage32.1 Intervention mean children- 2.0 Control mean children- 1.9 Intervention working %- 61.1 Control working %- 66.9	24.7 weeks	2 weeks	Standard website only	MVPA *via* survey MVPA *via* accelerometer- Increased in Intervention group in Questionnaire but not objectively *via* pedometer	MVPA survey Int- 202 min/week increase Cont- 110 min/week increase *p* = 0.027 *Via* accelerometer Int- 52 min/week increase Cont- 41 min/week increase *p* = 0.61	N/A	Mixed study with part digital in control. Not completely representative of normal socio-demographic group. Motivated cohort for exercise, so also may bias the results.
Albright et al., 2009^14^		Sedentary women n = 20 Mean age- 32.9 Working %- 45 Mean no. of children- 1.7	28 weeks	8 weeks	N/A	MVLPA *via* questionnaire	increased from 3 ± 13.4 minutes per week to 85.5 ± 76.4 minutes *p* < 0.001 Cohen’s d = 2.2 Effect size *r* = 0.7	PA barriers questionnaire Mean score at baseline 2.57 (1.4) Mean score at endpoint 2.31 (1.2) *p* < 0.03 80% satisfied with PA progress during intervention	No control group. Small sample size. Mixed intervention.
Bijlholt et al., 2021^16^		Normal postpartum population Intervention- 524 Control- 556 Intervention Mean age- 31.6 Control Mean age 32.	6 weeks	19.5 weeks	Conventional care	PA *via* IPAQ	MET mins- Median (Q1-Q3) Intervention- 1728 (920–3279) -2640 (1372–5397) Control 1857 (891–3438)-2461 (1342–4691) *p* = 0.40	N/A	Primary focus was on diet and eating behavior. Variation in usage of the app. Varied related to socio-demographic group. Significant drop out rate
Burkart et al., 2020^17^		At risk of gestational diabetes and CVD Intervention- 71 Control- 79 Mean Age- 27.7 No. of children 69–87.9% >1	6 weeks	46 weeks	Reduced education. No pedometer	Weekly MET levels from Questionnaire- no difference between groups	Change in weekly MET hours/week Intervention 4.9 (155.6) *p* = 0.89 Control 5.4 (124.0) *p* = 0.96	PA Self efficacy Intervention- 2.57 *p* = 0.04 Control- −0.96 *p* = 0.38 Difference between groups *p* = 0.31	Majority of the intervention was non-digital. Lack of blinding to intervention. Missing data so reduced sample size
Fjeldsoe et al., 2010^30^		Higher representation of low-level education, single parent and low-income households Intervention-45 Control- 43 Mean age- 28 >1 child- 59% Working %- 38%	Variable but <52 weeks	12 weeks	In person session and paper information pack	MVPA *via* questionnaire- frequency, duration Walking for exercise frequency, duration	Intervention frequency increased by 1.82 (0.18) versus control 0.24 (0.18) over intervention length *p* = 0.001 Walking for exercise freq increased by 1.08 (0.24) versus 0.73 (0.25) *p* = 0.004 Duration inc by 16.67 (13.3) versus 0.34 (13.64) *p* = 0.005	51% found the SMS/program extremely useful/useful	Small sample size. Mixed intervention. Differing exercise levels at baseline despite random allocation. Non standardised time post-partum
Khodabandeh et al., 2017^20^		Normal patient population Intervention- 112 Control- 108 Mean age intervention- 25.2 Mean age control- 24.2 Intervention working %- 20 Control working %- 12 No. of children- 1	0.86 weeks	6 weeks	Standard care	Self-reported PA- no difference	Walking Int 88 (83.8) Cont 92 (91.1) Moderate PA Int 16 (15.2) Cont 8 (7.9) Vigorous PA Int 1 (1.0) Cont 1 (1.0) *p* = 0.121	N/A	Commenced 6 days postpartum where PA is challenging. Intervention targeting dietary changes versus just PA. Significant in person element to intervention.
Lee et al., 2016^22^		Insufficiently active population Intervention- 30 Control- 29 Mean age Intervention- 33.1 Median no. of children- 1 Intervention % working- 6 Control % working −16	Mean 24–24.8 weeks	10 weeks	Educational leaflet provided	Objective MVPA and Questionnaire	Not improved- data not presented	N/A	No data presented on physical activity and how measured objectively versus control group. Small study size. Already Active population recruited- maybe different results in different pop. Study not purely digital
Van der Pligt et al.,^27^		Normal postpartum population Intervention- 28 Control groups- 48 + 43 Mean age- 32.4–33.2 Working %- 5–10 no. of children- 1–100%	39 weeks	39 weeks	Standard care	PA *via* questionnaire	No statistically significant difference between intervention and control Total PA difference between control 1–30.17 *p* = 0.606 between control 2–58.40 *p* = 0.278	N/A	intervention and control Total PA difference between control 1–30.17 *p* = 0.606 between control 2–58.40 *p* = 0.278N/A Inability to measure the usage of website and app.

IPAQ, International Physical Activity Questionnaire; MVPA, Moderate to vigorous physical activity; PA, physical activity.

**Table 4.** Intervention Details

Study ID	Mixed intervention or Full-Digital	Study design	Intervention
Kernot et al., 2019^19^	Full	RCT	3 arms: (1) 50-day Facebook team challenge + pedometer (4–8 women, 10,000-step goal, social support); (2) pedometer only; (3) control.
Kim et al., (2021)^21^	Full	Non-randomised experimental study	Mobile virtual-reality exercise program (123 motion-captured exercises).
Lewey et al., 2022^23^	Full	RCT	Wearable tracker sets baseline; virtual teams compete *via* a gamified app to exceed personalised step goals for points/levels.
Lim et al., 2021^24^	Full	RCT	Smartphone app logs weight/food/steps; web portal with dietitian, physiotherapist, and OT feedback/coaching.
Maturi et al., 2011^25^	Full	RCT	2-week tailored program encouraging increased walking using a pedometer-each woman wore a pedometer and recorded her daily step count, and was advised to increase their steps by 500 per week until they achieved the first target of 5,000 steps per day, continuing to increase it to minimum of 10,000.
Napolitano et al., 2021^29^	Full	RCT	Culturally-tailored smartphone app and private Facebook group. The app featured weekly content adapted from a diabetes prevention program, including lessons on healthy eating, stress management, and family activity, delivered *via* didactic videos and edutainment. Participants were sent motivational messages, set weekly diet and exercise goals, tracked their progress, and earned badges. The Facebook group fostered peer support and shared advice.
Albright et al., 2014^15^	Mixed	RCT	Motivational telephone counselling, pedometers, referral to community PA resources, social support, email advice on PA/pedometer goals, and newsletters
Albright et al., 2009^14^	Mixed	Pre-test post-test	17 telephones counselling calls with culturally matched counsellors, using motivational interviewing techniques. Access to a custom, mother-centric website. Participants were issued a pedometer to track and set step goals (10,000 steps/day). Provided tailored PA resources, directories, and newsletters
Bijlholt et al., 2021^16^	Mixed	RCT	Smartphone app lifestyle goal setting and tracking, motivational messages and tips, linked with activity tracker and weighing scale. This was complemented by four face-to-face coaching sessions using motivational interviewing, goal setting, action planning, and behavioural reinforcement techniques
Burkart et al., 2020^17^	Mixed	RCT	In person education sessions + pedometer and activity log
Fjeldsoe et al., 2010^30^	Mixed	RCT	2 in person sessions and 42 tailored SMS messages focusing on breaking social stigmas.
Khodabandeh et al., 2017^20^	Mixed	RCT	In-person education, information booklet, regular SMS, and phone counselling.
Lee et al., 2016^22^	Mixed	RCT	Two motivational consultations with a 10-week pram walking group, supported by goal-setting tools and a pedometer
Van der Pligt et al.,^27^	Mixed	Non-randomised experimental study	Online calorie tracking program, smartphone app, plus in-person education

RCT, Randomized controlled trial; SMS, Short message service (text).

### Data synthesis

Data were categorized to investigate the common types of digital technologies currently in use in assisting postpartum women return to PA, study type, and main outcome measures of PA used. Hybrid refers to a combination of digital strategies, with further detail seen in Table 5. In studies where changes in PA were displayed in sufficient detail, data were aggregated to look at mean changes in PA and differences between digital intervention and control groups. The period postpartum when the intervention began, and intervention length were also collated, along with their relationship to PA.

**Table 5.** Digital Technologies Used, Study Types, and Measures of PA

Digital technology used (*n*)	Study type (*n*)	PA measure (*n*)
SMS (*n* = 2)	RCT (10)	AWAS (1)
Applications (*n* = 3)	Pretest post-test (1)	AAS (2)
Pedometer (*n* = 3)	Feasibility (1)	IPAQ (5)
Hybrid (*n* = 6)	Non-randomized interventional study (1) Quasi-experimental(1)	Other questionnaires (6) Objective step count (5)

AAS, Active Australia Survey; AWAS, Australian Women’s Activity survey.

## Results

Search results yielded 1212 articles. Following screening of title and abstract, 120 articles had full text screening, with 14 articles eligible for inclusion in the review. The articles involved 2714 patients overall, with the most common digital interventions used being SMS text message,^18,20^ smartphone application,^16,26,27^, and activity trackers such as pedometers.^17,22^ A combination of these technologies was used in six studies.^14,15,19,21,23,24^

The most common strategies for measuring PA were patient questionnaires, with all 14 studies using some form of questionnaire as part of their data collection. Of these questionnaires, the most frequently used was the International Physical Activity Questionnaire (IPAQ),^31^ appearing in five studies. Objective step count data *via* pedometer or accelerometer was the second most common outcome measure, with five studies using this. Pedometers featured in the included studies both as an intervention—to display step count with a goal as a means of encouraging PA—and as an outcome measure to objectively record step count, highlighting their dual role in postpartum PA studies.

### Patient population

Ten of the studies included postpartum mothers who had developed or were at high risk of health-related problems as a result of inactivity or pregnancy. These comprised of physically inactive mothers in three studies (Albright, Maddock, and Nigg, 2009; Maturi, Afshary, and Abedi, 2011; Lee et al., 2016)^14,22,25^ with two studies investigating patients with gestational diabetes mellitus (GDM).^21,24^ Lewey et al.^23^ investigated patients with hypertensive disorders of pregnancy, and Burkart et al.^17^ with those at risk of cardiovascular disease or GDM. Fjeldsoe et al.^18^ studied low education and low-income households, Bijlholt et al.^16^ studied mothers with excessive pregnancy weight gain, and Napolitano et al.,^26^ with overweight mothers. Only 4 studies of the 14 were with a “normal” cohort of participants.

The average age of the participants ranged from 24.2 to 33.8, with a mean age (SD) of 30.02 (3.33) years across the 14 studies. The percentage of participants in a form of employment across studies varied from 5% to 81.5%, with participants having a variable no. of children (Table 3).

### Main findings

Of the 14 studies, 10 were RCTs, with the remaining four being a combination of feasibility, pre-test post-test, non-randomized, and quasi-experimental studies (Table 5). There were statistically significant improvements in seven studies on primary PA measure, with seven showing no statistically significant effect of the intervention. Fully digital interventions were present in six studies, while eight had mixed interventions (digital and non-digital). These mixed interventions suggest that digital tools are often used as an adjunct, complementing other strategies. For more details as to the nature of each intervention, see Table 4. Interventions included applications (mobile and virtual reality), advising PA regimens, wearable activity trackers, digital goal-setting tools, motivational online forums, and SMS messaging.

Where sufficient data were available in the studies between intervention and control, a percentage PA change across all groups was calculated (Table 6). Studies with inadequate data or with no control are not presented. The average increase in PA in the intervention groups was approximately 108% (SD 145.3), whereas in the control group it was 41.7% (72.5). The difference between the two groups was 65.9% (79.6). No formal statistical analyses have been applied to this meta-analysis, given the heterogeneity of the data. Three of the fully digital groups showed a significant increase in PA compared to their control, alongside two of the hybrid groups. For the subset of papers concerning fully digital interventions with adequate data (Table 6), the mean increase in PA is 65.5% (82.3) compared to the control group average of 22.6% (22.6). While percentage changes facilitate cross-study comparison, they should be read cautiously: baseline PA and postpartum starting points varied widely, so a large relative increase does not necessarily indicate that a woman has reached guideline-recommended levels. However, mothers start at very different activity levels and at different weeks postpartum, so calibrating to baseline removes some of that built-in inequality and lets you compare them on equal footing. Although participant-level calibration is methodologically sound, it does not in itself indicate whether women ultimately reach guideline-recommended activity levels; therefore, in future studies calibrated change should be reported alongside absolute post-intervention values and the proportion meeting established PA targets.”

**Table 6.** PA Outcome Measures, Changes, and Difference Between Groups

Study ID	Mixed intervention or Full-Digital	Outcome measure	Intervention change	Control change	Difference between groups
Albright et al.,^15^	Mixed	Overall MVPA minutes *via* survey	+459%	+239%	+220% *p* = 0.027
		Minutes of MVPA *via* accelerometer	+32%	+25%	+7% *p* = 0.61
Bijlholt et al.,^16^	Mixed	MET minutes *via* IPAQ questionnaire	+53%	+33%	+20% *p* = 0.40
Burkart et al.,^17^	Mixed	Weekly MET hours *via* questionnaire	+2.2%	+2.5%	−0.3% *p* > 0.05
Fjeldsoe et al.,^18^	Mixed	MVPA frequency *via* questionnaire	+101%	+14%	+87% *p* = 0.001
Kernot et al.,^19^	Full	Accelerometer derived MVPA	+29%	+9%	+20% *p* > 0.05
Kim et al.,^21^	Full	PA *via* questionnaire	+25%	+8%	+17% *p* < 0.001
Lewey et al.,^23^	Full	Difference in daily step count *via* pedometer	+26%	+16%	+10% *p* = 0.009
Maturi et al.,^25^	Full	IPAQ questionnaire for MVPA	+256%	+73%	+183% *p* = 0.001
Napolitano et al.,^26^	Full	MVPA *via* questionnaire	+121%	+22%	+99% *p* = 0.10
Van der Pligt et al.,^27^	Mixed	PA *via* questionnaire	+4%	+0.5%	+3.5% *p* = 0.28

Percent changes reflect relative improvements but may not indicate whether participants reached recommended levels of physical activity. Studies varied in postpartum timing and baseline PA, which limits comparability.

MET, Metabolic equivalent of task.

### Secondary PA outcomes

Secondary PA outcomes on perceived barriers, satisfaction, or self-confidence in returning to activity were investigated in 6 of the 14 studies. All six studies used self-reported questionnaires as measures for these outcomes. Burkart et al.^17^ and Napolitano et al.^26^ looked at PA self-efficacy (their confidence in knowing what and how to manage their PA). These showed no statistically significant differences between the intervention and control groups; these papers concerned mixed and fully digital interventions, respectively. Lim et al.^24^ used the health education impact questionnaire for reporting on health behaviors following its fully digital intervention, such as diet, exercise, and meditation, with behaviors aimed at promoting health or preventing illness. There was statistically significant improvement over the control group (*p* = 0.045). Albright et al.^14^ used a questionnaire to look at barriers to PA, such as time, self-consciousness about image, energy, weather, and family support and influence. While there was no control group, they reported a statistically significant reduction in barriers to PA with the hybrid intervention (*p* < 0.03). Fjeldsoe et al.^18^ and Kernot et al.^19^ investigated patient satisfaction *via* questionnaire, with the former reporting that 51% of the cohort found the hybrid intervention either extremely useful or useful in supporting return to PA and healthy behaviors. Kernot et al.^19^ reported 62% felt the app was of interest and 65% felt the app had assisted their increase in PA, although their quality-of-life questionnaire displayed no significant differences between the fully digital intervention and control groups.

### Intervention starting point and length

The average starting time was 14 weeks postpartum (SD 13 weeks) and ranged from 0 to 52 weeks postpartum. The mode and median lengths of intervention were 12 weeks, with five of the 14 studies using this timeframe. The mean length (SD) was 19.0 weeks (15.1). There was no apparent relationship between the type, timing, and duration of interventions.

## Discussion

This review investigated the use of digital health interventions and their value in supporting postpartum women back to PA. Many studies were with interventions targeted at populations at risk of pathology associated with pregnancy and inactivity.

There was some consensus on how PA was typically measured, with all the studies utilizing patient questionnaires looking at self-reported PA or moderate to vigorous physical activity (MVPA). Some of the studies used accelerometers or pedometers to objectively measure changes alongside self-reported results, although there is consensus that questionnaires such as the IPAQ^31^ and Australian Women’s Activity survey^30^ are reliable and valid measures. Data about digital interventions were collated to gauge current usage and offer direction on future interventions and studies.

An equal number of studies showed a statistically significant improvement in PA, versus those that didn’t, versus control. Secondary outcomes of the study assessed measures such as patient satisfaction and confidence in returning to PA. These were predominantly measured by patient questionnaires with mixed results, although there was certainly evidence of value, satisfaction, and perceived effectiveness in 4 of the 6 interventions.

Of the studies that did report data on PA versus control (Table 6), there were large changes in PA, with an average of 65.9% difference between groups, suggesting an opportunity to improve return to PA. Although percentage increases are useful for cross-study comparison, they must be interpreted carefully, as due to varied starting points, a large relative gain does not necessarily mean participants achieved guideline-recommended activity. We therefore recommend that future studies report absolute post-intervention PA and the proportion of women meeting established guidelines.

One important concept the authors highlight is that there is no “one size fits all” approach to creating a successful postpartum intervention. For example, consider a working mother whose local healthcare service offers postnatal appointments only on specific days and times—a setup that may not fit her schedule. Someone with a smartphone who is familiar with using apps and activity trackers may find digital tools particularly valuable, whereas another individual with easier access to in-person services, or without a smartphone, may not benefit in the same way. However, it’s important to avoid framing this as a simple either/or choice. Digital interventions should not be seen as a replacement for in-person or conventional strategies, but rather as a modern complement that expands and enhances the options available to postpartum women.

This also correlates with work by Western et al.,^32^ which found that digital interventions were more effective in higher socioeconomic groups. Several of the studies in our systematic review looked at low PA groups and low socioeconomic and education groups,^16,18,22,25^ answering the question of whether results in digital interventions accurately represented in these cohorts, despite barriers to access and digital inequality. Bijlholt et al.^16^ also displayed less utilization in the lower socioeconomic groups with significant dropouts, evidencing the need for targeted cohorts and digital equality.

Further formal statistical analyses were not possible due to the heterogeneity of the studies. Due to the scarcity of studies looking purely at digital intervention and PA, several of the studies were investigating numerous outcomes from their interventions, such as diet, and weight gain or loss, with PA often being a secondary outcome measure, with less author or intervention focus and content targeted at PA.^16,21,22,24^

There was also a wide variety in start dates of the intervention and intervention length, with some studies starting immediately postpartum and others at almost 12 months postpartum. Due to the nature of postpartum physiology, PA in the very early stages has extra barriers, such as pain, fatigue, and time challenges. As a woman recovers from the physical effects of giving birth, PA is possibly more likely to improve organically. However, the studies did not display great effectiveness at any specific start point. Consideration of the optimal starting point for an intervention should be a priority for future studies in this cohort. Again, there was also a wide variety in the intervention length, however, it does appear that a 12-week intervention period is the most utilized across the studies, with 4 of this length being effective at increasing PA,^18,21,23,25^ versus one^26^ being ineffective compared to control.

Further limitations of some studies were that the control groups also had some digital intervention as part of the study.^14,15,23^ While there was increased digital content in the intervention groups, this also provided challenges in interpreting results purely as digital versus conventional (non-digital) strategies. Mixed interventions were present in eight of the studies, which included face-to-face and conventional delivery.^14–18,20,22,27^ This does make it challenging to conclude which part of the intervention was responsible for the outcomes.

Interventions labeled as hybrid have digital and non-digital aspects. Due to the overlap, it is not possible to gauge the individual effect of digital technologies in these studies. However, hybrid approaches may more accurately reflect how digital tools are adopted in real-world settings, supplementing care under the status quo. Although clear stratification would clearly display attribution of effect, our categorization gives insight into the use of digital interventions as part of wider strategies, while highlighting the need for more focused research on the specific added value of digital interventions.

Although this review found seven articles displaying an effect and seven articles displaying no significant effect versus control, there is still a case for using digital health interventions in the current climate. A comparison of studies that demonstrated statistically significant improvements in PA versus those that did not reveal several distinguishing features. Effective interventions tended to include interactive elements such as personalized goal setting (*e.g.,* Kim et al., Lewey et al.), gamification or team-based motivation (*e.g.,* Lewey et al., Fjeldsoe et al.), and consistent self-monitoring (*e.g.,* Maturi et al.). These studies also had longer durations (typically 12 weeks) and often included digital tools with behavioral reinforcement. In contrast, ineffective interventions were more likely to feature passive content (*e.g.,* educational apps or written materials), shorter durations, or early postpartum delivery when women may be less physically able to engage (*e.g.,* Khodabandeh et al., Napolitano et al.). However, some heterogeneity persists—for example, studies with similar tools like smartphone apps showed varying results, suggesting context, engagement, and user tailoring may also be key determinants. Future trials should prioritize interactivity, sustained delivery, and tailoring to postpartum readiness for optimal outcomes. There is increasing access to digital technology, although digital inequality must be considered in health strategies moving forward to ensure equity and fairness.^29^ We would suggest either targeting cohorts of higher socioeconomic status or ensuring participants have digital access, and the means to use it.

However, we are also at a time where NHS waiting lists are long, and accessing in-person services is difficult.^33^ While further research is required, digital or e-health strategies in other conditions have been shown to be as effective as conventional care, with reduced costs.^34,35^ It also forms a key part of the NHS Long-term plan.^8^ In this context, there is value in looking into greater usage, albeit with more focused studies to support those decisions in postpartum care. Walker et al.^9^ have displayed the effectiveness of a smartphone application for musculoskeletal conditions. This type of application provides educational content, reminding “nudges”, exercise videos, goal planning, and links to local providers, such as social groups, support groups, and exercise classes. When considering the barriers and enablers to PA in pregnant and postpartum women identified in the introduction, this type of intervention could go some way to addressing a number of these. Clearly, there is the initial cost of creation of content, ongoing technical support, and roll-out; however, a large-scale study using simple interventions, with simple outcome measures, could provide cost analysis data for large organizations such as the NHS.

In developing further studies, the patient cohort must also be carefully considered. Is the aim or best use of digital strategies to target the masses to improve population health and reduce the burden on healthcare providers? Or is there a place in the sporting world to enhance support for postpartum women to return to their chosen activity? If purely interested in comparing digital intervention versus conventional, future studies are recommended to include a purely digital intervention and a non-digital control group to make more accurate conclusions. Thought must also go into the environment implementing these changes, the cohort and the intervention must be specific, with sufficient effect size, and this is lacking in the current literature.

## Conclusion

There is sufficient promise within the evidence found in this review to recommend further targeted trials into digital health interventions for postpartum women to aid their return to PA. Relatively inexpensive technologies such as smartphone applications, activity trackers, and text messages are the most utilized technologies, with simple outcome measures such as step count and questionnaires. Cohort accessibility to such technologies is likely to be high, although care must be taken to ensure digital equity, targeted groups, and environments. The current health care environment must also be considered, where waiting lists and access to face-to-face care are certainly difficult. While this may not be an intervention that works for all, it has sufficient promise to be effective for a significant portion, with a 12-week intervention length appearing effective. A large-scale study using simple interventions could make it the new standard of care, aligning with the NHS Long-term plan, and inspire a digital rollout for other conditions, with PA being effective as a preventative measure from disease and proactively supporting long-term health. Further studies into elite athlete use are also recommended, with digital technology likely to be utilized as part of their return to sport process, albeit not yet published.

## Abbreviations Used

GDM: Gestational diabetes mellitus
IPAQ: International Physical Activity Questionnaire
MVPA: Moderate to vigorous physical activity
PA: Physical activity
